# Mechanism analysis of rural residents' participation in green energy transition: A community-level case study in Nanjing, China

**DOI:** 10.1016/j.heliyon.2024.e33951

**Published:** 2024-07-02

**Authors:** Ling Li, Jintu Gu, Di Wu, Abdul Rasool khoso

**Affiliations:** a Department of Sociology, Hohai University, China; b Research Center for Environment and Society, Hohai University, China; c Jiangsu Research Base of Yangtze Institute for Conservation and High-quality Development, China

**Keywords:** Green energy, Rooftop photovoltaic, Embedding theory, Threshold model, Self-funded rural residents, China government policies

## Abstract

Achieving a transition to green energy requires the government to adopt new policies on green industrial products and energy. Throughout this process, rural residents often face various challenges due to economic and other factors. However, some rural residents are motivated to participate in this transition due to the economic benefits of energy usage and rooftop photovoltaic (PV). This study takes a rural community in the outskirts of Nanjing, China, as an example and applies Granovetter's embeddedness theory and a threshold model to analyze the factors influencing rural residents' engagement in a fair transition to green energy. Research hypotheses are proposed accordingly. The results indicate that rural residents are influenced by multiple factors in the adoption process of rooftop PV projects, primarily encompassing economic and trust-related aspects. From an economic perspective, rural residents evaluate the viability of rooftop PV systems by considering the marketing strategies employed by PV enterprises and the governmental pressure to reduce carbon emissions. They make rational calculations to determine the return on investment, and only when the economic threshold is surpassed will they reach the anticipated level of participation. From the perspective of trust, rural residents' participation in rooftop PV projects is also influenced by trust factors. The level of trust that rural residents have in rooftop PV enterprises, governments, and community organizations plays an important role in their willingness to participate in the green energy transition. Based on these findings, the research paper concludes that local government should continue providing tailored public information and services to facilitate the progress of rooftop PV projects.

## 1 Introduction

On September 22, 2020, the Chinese government proposed carbon peaking and carbon neutrality at the 75th session of the United Nations General Assembly [1]. Green transformation of energy structure is one of the important grasps to achieve it [2]. The green transformation of the energy structure in rural areas is of great significance to achieving the Sustainable Development Goals, addressing global climate change and energy security challenges and requires the joint efforts and cooperation of the international community. However, Rural residents are considered to be at a disadvantage in the energy transition. There exist rural residents who want to participate in it based on their energy use status and economic benefits. In this process, rural residents' participation is considered a key factor in achieving a fair transition. However, rural residents face numerous difficulties and challenges that limit their active engagement in the transition to green energy.

Green energy transition is one of the key measures to address global climate change and energy security challenges. Globally, as the environmental pollution and resource depletion caused by over-reliance on traditional fossil fuels become increasingly prominent, governments and international organizations have stepped up their efforts in the research and application of green energy. China, as one of the world's largest energy consumers, has significant global influence in restructuring its energy structure and transitioning to green energy. In a global context, breakthroughs in green energy technology continue to be made, including various forms of renewable energy such as solar, wind, and hydroelectric power. In particular, the cost of solar and wind energy technologies continues to decrease, making them major sources of sustainable energy. However, the global green energy transition still faces a series of challenges, encompassing technological costs, energy storage, grid construction limitations, as well as imperfect policy and market systems. Meanwhile, climate change issues are intensifying globally, necessitating an accelerated pace of green energy transition. In China, with the introduction of national strategies such as "carbon peaking and carbon neutrality," green energy transition has become one of the country's important development directions. The Chinese government has increased policy support and investment in renewable energy, promoting rapid development in areas such as solar and wind energy. As of now, China has become the world's largest solar and wind energy market, achieving significant accomplishments. Nevertheless, China's green energy transition also faces numerous challenges, involving the complexity of energy structure adjustment, slow progress in power system reform, and local protectionism issues. Additionally, as one of the major sources of global carbon emissions, China is facing increasing pressure to address climate change, necessitating an accelerated pace of green energy transition to achieve a win-win situation between economic development and environmental protection. Therefore, green energy transition is an inevitable choice to cope with the challenges of climate change and energy security. Moreover, it also is one of the key paths to promoting sustainable economic development and achieving environmental protection goals. Strengthening international cooperation, increasing innovation and application of green technologies, improving policies and market mechanisms, and promoting the smooth progress of green energy transition are of great significance to the realization of sustainable development goals.

As an important project of the green energy transition in rural areas, photovoltaic (PV) power generation can provide a clean and sustainable energy supply for rural areas and promote economic development and social progress in rural areas. To explore in-depth the mechanisms behind rural residents' participation in a transition to green energy and provide targeted guidance for policymakers and practitioners, this study conducted an in-depth investigation using the rooftop PV project in the L Community, located in Nanjing, China, as a case study.

There are two significant reasons for selecting Nanjing as the research site. Firstly, As one of the important cities in China, Nanjing's rooftop PV power generation projects are representative in geographical location. Through the case analysis of the Nanjing rooftop PV power generation project, we can better understand the implementation process and effect of energy-green transformation. Second, rooftop PV power generation technology is a globally shared technology, and its application benefits from cooperation on a global scale. The Nanjing rooftop PV power generation project draws on successful cases and practices from other parts of the world to promote technological innovation and improve project efficiency. By conducting an in-depth analysis of the mechanisms of rural residents' participation in the rooftop PV project in the L Community, this study can better understand the nature of these challenges and provide feasible solutions to drive a transition.

The experiment delves into the mechanisms of rural residents' participation in a transition in Nanjing, identifying key factors and barriers that may influence their participation. Based on the research findings, this study proposes policy and practice recommendations to facilitate the broader engagement of rural residents in the transition to green energy. This experiment ensures the sustainability and inclusiveness of the transition, allowing all community members to share the economic and environmental benefits brought about by the transformation to green energy.

This study comprises five sections. Section 1 is the introduction, which discusses the importance of green energy transition under the challenges of global climate change and energy security, as well as the "carbon peak and carbon neutrality" strategy proposed by the Chinese government. It emphasizes the crucial role of rural areas in the green transformation of energy structure and identifies the challenges faced by rural residents in participating in this process. Section 2 is the literature review, which reviews research on rural residents' participation in green energy transition globally, covering factors such as economic incentives, government policies, social trust, and technological innovation. It points out the deficiencies in existing research, such as the lack of a comprehensive understanding of the impact of multiple factors, insufficient discussion on the applicability of research results in different contexts, and limited exploration of research method application and limitations. Section 3 is the research methodology, including case selection, data collection, and an explanation of analyzing the factors influencing rural residents' participation in green energy transition based on a threshold model. Section 4 presents the research results, analyzing the types and quantities of rooftop photovoltaic installations by rural residents, as well as their sources of information and types of trust. Meanwhile, this study investigates the degree of rural residents' participation in the green energy transition, their motivations, and interests. Moreover, it discusses the level of trust rural residents have in different sources of information, and the obstacles influencing their participation in the green energy transition. Section 5 is the conclusion and discussion, which investigates the impact of dual economic and trust thresholds on rural residents' participation in green energy transition. It emphasizes the importance of government subsidy policies, market trust, and government trust, and proposes targeted policy recommendations. In addition, it summarizes the influence of rural residents' knowledge levels, government policy support, and social networks on their participation in green energy transition, supporting the research hypotheses proposed earlier. The entire research framework aims to comprehensively understand the mechanisms influencing rural residents' participation in green energy projects by considering multiple factors such as economic incentives, government policies, social trust, and community networks. Furthermore, more accurate and effective strategies are proposed to promote fair and sustainable energy transition in rural areas.

## 2 Literature review

In the global context of the transition to green energy, the participation of rural residents in the green energy transition has become an important issue. Many researchers have looked at various factors in this process, including economic incentives, government policies, social trust, and technological innovation. These studies acknowledge the significance of long-standing research in innovation diffusion for understanding the adoption process of new technologies and concepts in rural communities. Economic incentives are widely recognized as a key factor in enabling rural residents to participate in the green energy transition. For example, Zhang et al. (2023) analyzed the economic feasibility of solar PV projects in China's rural areas. They pointed out that when the government provided sufficient financial subsidies, solar projects could achieve a faster return cycle, thus attracting the participation of rural residents [3]. However, these studies often overlooked other influencing factors beyond economic incentives, such as the role of culture and social trust. Regarding the impact of government policies, Mohazzem Hossain et al. (2024) discussed the role of government in promoting rural green energy projects. They found that government policy support and regulatory pressure were crucial to the success of solar energy projects in rural areas [4]. However, most of the studies in this field remained at the macro level and lacked in-depth research on the perception and reaction of rural residents. Social trust was also vital for rural residents to participate in green energy projects. Mustafa et al. (2023) showed that individuals' trust in the government and technology suppliers significantly affected their willingness to accept new energy technologies [5]. Despite this, existing research often failed to adequately consider the impact of social networks and social capital within communities on trust levels, which was important for understanding and promoting the participation of rural residents. Furthermore, they recognized the critical role of the government in policy formulation and support provision, as well as the impact of market factors on rural residents' acceptance of green energy solutions. Firstly, as users of green energy, the behavior and willingness of rural residents undoubtedly have a meaningful impact on the transition to green energy in rural areas. Rural residents' attitudes towards green energy are influenced by the innovation diffusion theory, which explores how new technologies spread within society and focuses on the adoption and diffusion of innovations. In the case of rural residents, the availability, ease of use, and performance of green energy technologies are vital considerations for their acceptance [6,7]. Additionally, information exchange, social networks, and neighborhood influences among rural residents may also impact their attitudes [8]. Therefore, research needs to focus on rural residents' awareness and knowledge levels of green energy technologies, their channels for acquiring information, and the influence of social interactions on the adoption of green energy.

Secondly, it is the government's responsibility to oversee the management of green energy, and their policies play a crucial role in shaping the acceptance of new energy technologies among rural residents [9]. The government typically implements measures such as subsidies, tax incentives, demonstration programs, and public awareness campaigns to support rural residents' adoption of green energy technologies and enhance their knowledge in this domain [10]. Notably, some scholars contend that satisfaction with government policies significantly influences the behavioral adoption of new energy technologies among rural residents [11]. Asif et al. (2023) focused on the usage of solar panels by consumers in Pakistan. They examined the associations between value orientation, utilitarian benefits, collectivism, adoption reasons, attitudes toward renewable energy, and adoption intentions [12]. Their study revealed that value orientation, utilitarian benefits, collectivism, and adoption reasons positively and significantly impacted Pakistani consumers' attitudes and intentions to adopt renewable energy. This aligned to some extent with this study's findings regarding the influence of economic factors and trust factors on rural residents' participation in the green energy transition. Hailiang et al. (2023) explored the impact of green finance and renewable energy on the Chinese tourism industry, considering technological progress, health expenditures, and carbon emissions. Their research found a positive correlation between renewable energy, green finance, and technological innovation in the tourism industry, while health costs and carbon emissions significantly reduced tourism activities [13]. Additionally, the moderate effects of renewable energy and green finance led to a significant growth in tourism activities. Moreover, panel causality analysis demonstrated robust causal relationships among the selected variables. Wong et al. (2024) focused on the adoption intentions of renewable energy in Malaysia, aiming to identify key factors shaping individuals' willingness to use renewable energy [14]. They utilized partial least squares structural equation modeling, finite mixture partial least squares, and importance-performance map analysis to analyze the collected survey data. The results of their study indicated that the cost, usability, and accessibility of renewable energy remained limiting factors for adopting renewable energy in Malaysia. Government policies and support measures directly impact rural residents' attitudes and behaviors toward green energy. The government can promote the acceptance and adoption of green energy among rural residents by implementing incentive policies, providing financial support and subsidies, establishing regulatory standards, and conducting promotional and educational activities. Active government involvement and support can increase rural residents' awareness of green energy, alleviate economic burdens, and provide feasible green energy solutions for them.

Finally, the market assumes the role of a supplier of green energy, and enterprises are tasked with enhancing their operational efficiency, prioritizing green and high-quality development objectives [15,16], and reducing product prices [17], while also fulfilling their social responsibilities. Dzwigol et al. (2023) focused on green economic growth in European Union (EU) countries, aiming to explore the impact of environmental regulations, renewable energy, and energy efficiency [18]. The results confirmed the significance of renewable energy, environmental regulations, and energy efficiency in green economic growth, proposing relevant policy recommendations. This study, along with Dzwigol's research, was dedicated to investigating the influence of environmental regulations, energy efficiency, and renewable energy on green economic growth, showing some similarities in methods and data analysis. Therefore, the results of these two studies can be mutually referenced, providing crucial policy suggestions and guidance for EU countries to achieve green economic growth. Given that the primary cost lies in information dissemination [19], enterprises can effectively communicate green energy-related information to rural residents [20], thus ensuring the safety and reliability of green energy products. Luo et al. (2024) highlighted the asymmetric impact of information and communication technology (ICT) on energy transition. The study found that the influence of ICT on green energy production exhibited asymmetry across different countries and situations [21]. For instance, in countries such as Canada, France, Italy, Japan, and the United Kingdom, the installation of ICT devices can facilitate the energy transition process, especially in low to high quantiles. However, in countries like China, Germany, Spain, and the United States, the impact of ICT on green energy production had both promoting and inhibiting effects across different quantiles. Additionally, the study employed continuous wavelet transform causality tests to validate the reliability of quantile regression estimation results. The development of the market, competition, and price trends directly impact the availability and affordability of green energy products. Additionally, rural residents' consumer preferences and demands also influence their choices and acceptance of green energy. A diversified product selection and viable economic models provided by the market can meet the needs of rural residents and offer cost-effective and sustainable green energy solutions.

The green energy transition is a complex process involving various factors such as policy, technology, society, and economy. In the formulation and implementation of green energy policies, scholars have conducted extensive research. Ainou et al. (2023) analyzed the energy security situation in Morocco from 2000 to 2016, This study suggested the integration of a higher proportion of renewable energy into the energy structure and promoting the development of efficient technologies through large-scale green finance and investment projects [22]. Ma (2023) explored the potential for energy transition under current technological conditions by analyzing green productivity and renewable energy development goals in 40 industrialized countries from 1990 to 2020. This research was crucial for understanding the progress and challenges of industrialized countries in green energy transition [23]. It emphasized the pivotal role of technological advancement in driving dual improvements in the economy and the environment and highlighted the positive impact of trade in promoting green output. These findings offered valuable references for formulating and implementing green energy policies, while also providing insights for cooperation and economic transformation among industrialized countries. Ma et al. (2024) provided empirical analysis on the specific role of green energy innovation in the energy transition of G7 countries, examining the impact of green energy innovation on the overall national energy transition from a macro perspective [24]. Additionally, the Chinese government introduced a series of policies to support the development of green energy, such as the Renewable Energy Law, and the Renewable Energy Development Plan, as well as various subsidies and preferential policies. The formulation and implementation of these policies played a significant role in promoting green energy transition. However, there were also issues such as inadequate enforcement and frequent policy adjustments that should be further strengthened and improved. Regarding the factors influencing community participation in green energy transition, scholars focused on various aspects including social, economic, and institutional factors. For instance, Trevisan et al. (2023) integrated renewable energy communities into Italy's regulatory framework to achieve community climate neutrality goals and pursue the ecological and energy transition objectives specified in the national recovery and resilience plan [25]. Okoli et al. (2024) examined the sustainable energy transition strategies in the petroleum industry, comparing comprehensively the United States and the African petroleum industry. This study offered valuable insights for policymakers, industry leaders, and stakeholders to promote the sustainable energy transition in the petroleum industry [26]. The social capital theory posits that trust relationships, social networks, and cooperation mechanisms within communities play crucial roles in driving the success of green energy projects. The technology acceptance model focuses on individuals' cognitive processes and acceptance of new technologies, highlighting the impact of factors such as publicity, education, and information transparency on community participation in green energy transition. In addition to these, some other theories and models can be utilized to explain the dynamic process of community involvement in green energy transition, such as socio-economic theory, political ecology, etc. These theories and models provide a theoretical foundation and analytical framework for a deeper understanding of green energy transition. In China, the latest green energy policies and practical cases are also worth noting. For example, the Chinese government has set targets for carbon peaking and carbon neutrality, increasing policy support for green energy. Meanwhile, some local governments are actively exploring ways for community involvement in green energy transition, such as building community solar projects and promoting energy sharing. These practical cases provide valuable experience and insights for further research on rural community participation in green energy transition. In summary, the green energy transition is an intricate and vital issue that requires comprehensive consideration of policy, technology, social, and economic factors. By applying various theories and models, the dynamic process of green energy transition can be comprehensively understood and analyzed, furnishing theoretical support and policy recommendations for promoting green energy transition.

Existing studies, when analyzing the motivations behind rural residents' participation in the green energy transition, often tend to consider individual factors in isolation, lacking a comprehensive perspective to understand the compound factors influencing participation comprehensively. Additionally, most research focuses on specific technologies or policy solutions, neglecting the role of factors such as local culture, community structure, and resident interactions in the decision-making process. This has resulted in a limited understanding of how to promote green energy transition in rural areas effectively. Firstly, in the review of existing research, some factors such as economic incentives, government policies, and social trust affecting rural residents' participation in green energy transition are mentioned, but there is a lack of in-depth exploration of the interactions and complex relationships among these factors. For example, there may be mutual influences between economic incentives and social trust, and how government policies affect rural residents' participation under diverse economic and social backgrounds also deserves deeper investigation. Therefore, future research could attempt to comprehensively understand the integrated impact of these factors on rural residents' participation in the green energy transition by integrating multiple factors and adopting more sophisticated analytical methods. Secondly, although some relevant studies in China and other countries are introduced, there is limited discussion on the applicability and generalizability of these research findings in different contexts. Considering the differences in policies, cultures, and economies among various countries and regions, it is necessary to further explore whether there are significant differences in the impact of these factors on rural residents' participation in the green energy transition and to propose corresponding customized strategies and recommendations. Additionally, although some research methods and data analysis techniques are presented in this study, there is relatively less discussion on the application and limitations of these methods in specific research. Different research methods may have various impacts on research results, so it is necessary to delve deeper into the advantages and disadvantages of diverse methods and how to select and combine these methods to enhance this study's credibility and effectiveness. Finally, although some policy suggestions and practical experiences are mentioned, there is limited evaluation of the feasibility and implementation effects of these suggestions and experiences. When proposing policy suggestions and promoting practices, it is essential to consider more practical situations and feedback from stakeholders to ensure that these suggestions and practices can achieve sustainable outcomes. Consequently, based on the above analysis, this study proposes the application of Granovetter's embeddedness theory and threshold models to analyze the mechanisms behind rural residents' participation in the green energy transition, addressing the shortcomings of existing research. By comprehensively considering multiple factors, encompassing economic incentives, government policies, social trust, and community networks, this study aims to gain an in-depth understanding of the comprehensive mechanisms influencing rural residents' participation in green energy projects. It seeks to propose more precise and effective strategies to promote fair and sustainable energy transition in rural areas.

## 3 Research methodology

### 3.1 Case selection

Green energy, also referred to as sustainable energy, pertains to environmentally friendly sources of power that have minimal impact on the environment, require low resource consumption, and possess extensive application prospects. Given the context of global warming and escalating environmental pollution, the research and implementation of green energy have become a focal point in China and worldwide. Solar energy, wind energy, hydroelectric power, biomass energy, geothermal energy, and other renewable sources comprise green energy. These forms of energy are characterized by their renewability, cleanliness, and low carbon emissions levels, thus making them a crucial choice for promoting optimal energy structures and reducing carbon emissions.

Among the various green energy, solar energy has emerged as a focal point for development due to its clean, pollution-free, and inexhaustible advantages. As a crucial means of harnessing solar energy, PV power generation has garnered widespread attention and application. The rooftop PV has become a research hotspot owing to its unique advantages within this domain. The rooftop PV involves installing PV modules on building roofs to directly convert solar energy into electricity. Compared with other PV power generation systems, rooftop PV power generation systems offer high space utilization, convenient grid access, significant environmental benefits, and remarkable economic advantages.

**Fig. 1:**
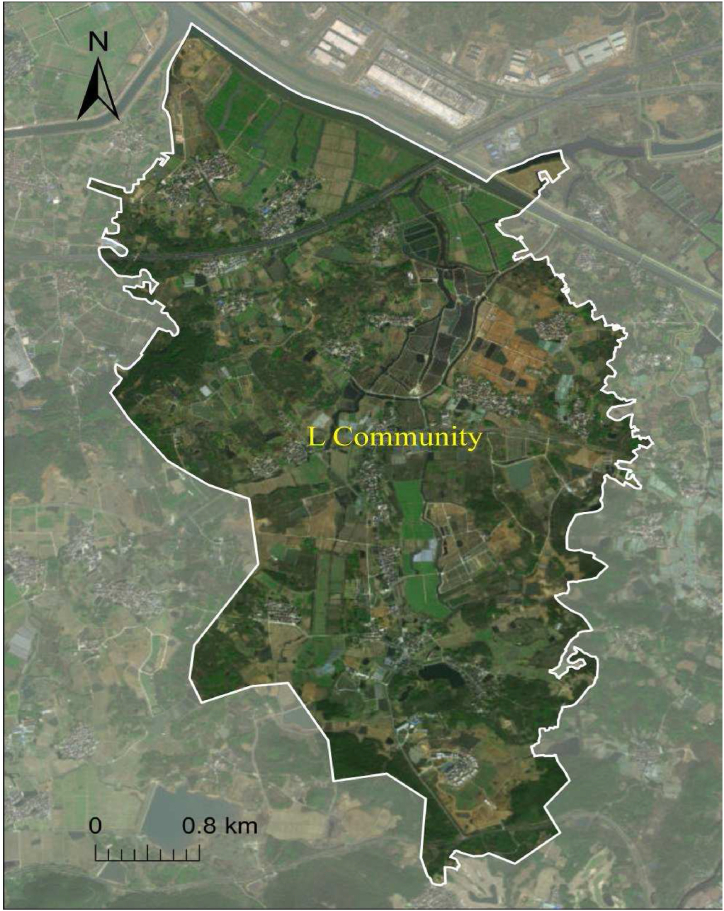
The map of the approximate location of the surveyed community.

As the capital city of China, the rooftop PV project of Nanjing reflects the development status and trend of China's green energy transition to a certain extent. At the same time, the application of rooftop PV power generation technology benefits from international cooperation and experience sharing. The rooftop PV projects in Nanjing benefit from the experience of other parts of the world, which drives the continuous innovation of the technology and the improvement of project efficiency. Therefore, this study takes the rooftop PV project of the L community in Nanjing as a case, as depicted in Fig. 1.

**Fig. 2:**
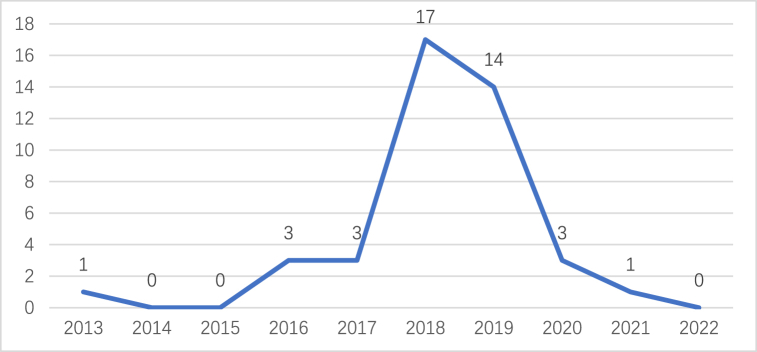
Change in the number of rooftop PV installations in the L community.

The L community spans an area of 18 square kilometers and is situated approximately 50 km from Nanjing. It comprises 17 natural villages and accommodates a population of 4559 residents residing in a total of 1295 households. Information of residents is exhibited in Table 1. The initial adoption of rooftop PV systems within the L community occurred in 2013, with subsequent years witnessing a surge in installations, particularly in 2016. Over a span of ten years, from 2013 to 2022, a cumulative total of 42 rooftop PV installations were recorded, as indicated in Fig. 2.

**Table 1:** Resident information in the L community.

Variable		Number
Gender	Male	2301(50.47 %)
	FeMale	2258(49.53 %)
Age	<26	489(10.73 %)
	26–40	1099(24.11 %)
	41–55	1291(28.32 %)
	56–70	1410(30.93 %)
	>70	270(5.91 %)
Education	Low	567(12.44 %)
	Medium	3568(78.26 %)
	High	424(9.30 %)

Case interviews and field observation are used to collect data. Case interview is an essential method to get a deeper understanding of individuals, organizations or events. Through communication and communication with interviewees, rich qualitative data can be obtained to provide in-depth understanding and insights for research. PV projects face special challenges in ensuring data integrity and accuracy. Because it involves many aspects of data, including technical parameters, economic indicators, PV information sources, etc. Therefore, this study establishes a unified data collection standard and process, and each interviewee is interviewed by a fixed researcher. At the beginning of March 2022, the researchers began preliminary contact with residents in the community who had installed rooftop PV and those who had not installed them, and formal research was launched in April. The initial phase of interviews encompassing all residents within the community who had implemented rooftop PV systems was concluded between July and August. Subsequently, intermittent visits were made to the community to conduct in-depth interviews with select residents, thereby supplementing the data required for the survey. This study design allows for a comparison between residents who have already installed rooftop PV and those who do not understand the motivations, barriers, and attitudes of rural residents regarding their participation in a transition to green energy.

In field observation, the researcher goes directly to the site where the observation object is located and obtains the data through detailed records. To ensure the successful execution of the study, the researcher initiated contact with the community secretary in early March 2022 to discuss the research objectives. Subsequently, the community secretary provided a comprehensive list of residents who had installed rooftop PV in each natural village. Accompanied by the village accountant, the researcher conducted field interviews within the residents' homes. This research approach integrated direct observation and case interviews, ensuring a comprehensive data collection process. Data for the study were obtained through various sources.All participants have granted their consent for interviews and participant observations to be conducted by the research team.

### 3.2 Analysis of factors influencing rural residents' participation in the transition to green energy based on the threshold model

Due to the constraints of the rural residents themselves, as the rational people in the market, rural residents comprehensively consider rooftop PV inputs and economic benefits. Inputs involve economic costs, and rooftop PV is a long-life product, the sustainability of return benefits based on its regular and safe operation. However, it also needs to invest time in essential operation and maintenance [27].

For instance, within Korean rural communities, women tend to exhibit hesitation in adopting innovations until a certain percentage of women within the same community have embraced them, as their comfort level is influenced by social validation [28,29]. In studies examining migration intentions, the decision to migrate is strongly influenced by other migrants, a phenomenon referred to as "chain migration," and individuals with lower thresholds tend to possess greater psychological and economic resources [30]. In collective behavior, different actors exhibit varying thresholds for engaging in the same practice. The concept of "embeddedness" was initially introduced by Polanyi [31] in economic theory, highlighting the need to explore the social relations in which individuals and organizations are embedded. Granovetter proposed that economic behavior was embedded within social structures, with social networks as the core structure and trust being the network mechanism facilitating embedding [32]. The acceptance process of green energy among rural residents constitutes an economic behavior. According to Granovetter's embedding theory, economic behavior and social trust networks are mutually embedded. Consequently, rural residents encounter economic and trust thresholds throughout this process.

Threshold models also referred to as threshold effects, are frequently employed in disciplines such as Economics, Statistics, and Management [33]. This modeling approach serves as a valuable method for data analysis, particularly in the investigation of threshold effects or nonlinear relationships among various influencing factors. It aids in comprehending the associations between different variables and identifying the circumstances under which these relationships change. When employing the threshold model for data analysis, it is imperative to initially define relevant variables and formulate relevant hypotheses. 1. Create variables. The primary dependent variable involves the degree or attitude of rural residents towards participating in the equitable transition to green energy. Independent variables encompass factors potentially influencing rural residents' participation in the fair transition to green energy, such as knowledge level, economic conditions, government policy support, and social networks. Building upon the current research landscape, the following research hypotheses are posited.

Hypothesis 1 A positive correlation exists between the knowledge level of rural residents and their active participation in the equitable transition to green energy. Individuals with elevated knowledge levels are likely to demonstrate a greater inclination to engage in the transition to green energy.

Hypothesis 2 There is a positive correlation between government policy support and the degree of rural residents' participation in the fair transition to green energy. The more comprehensive the government's policy support, the higher the motivation for rural residents to actively participate in the transition to green energy.

Hypothesis 3 Social networks exert an influence on the degree of rural residents' participation in the fair transition to green energy. Rural residents with extensive social networks may find it easier to access information and resources, thus actively participating in the transition to green energy.

### 3.3 Economic analysis of rooftop PVs for rural residents

In addition to considering natural conditions, such as geographic light resources, the economic benefits of rooftop PV are influenced by three factors: PV cost, PV subsidy, and household self-consumption rate, which can be theoretically calculated. The cost of rooftop PV installations has decreased as market technologies mature. According to a 2022 report by the International Renewable Energy Agency (IRENA) on the cost of renewable energy generation, the cost in China decreased from 20.18 yuan per watt in 2012 to 4.04 yuan per watt in 2021, representing an 80 % reduction over ten years [34]. In the L community, companies began promoting rooftop PV installations in 2016, and the cost of PV installations decreased from 12 yuan per watt in 2016 to 3.8 yuan per watt in 2021, maintaining this price in 2022. The per-watt cost for rooftop PV installations in the L community is marginally lower than the national average. Furthermore, the rooftop PV subsidies in the L community align with national standards without any additional subsidies provided at the provincial, municipal, or district levels.

Subsidies denote the economic support measures enacted by the government to incentivize rural residents to participate in the transition to green energy. These subsidies are usually provided in the form of financial subsidies, electricity price discounts, or tax relief. Government-set subsidy standards are generally based on factors such as the installed capacity, electricity generation, or investment costs of PV power generation. Specific subsidy amounts or proportions are determined through certain calculation methods.

#### 3.3.1 Rooftop PV costs and subsidies

In the L Community, rooftop PV systems are installed in 40 households with a total installed capacity of 8 kW. Out of these, 35 households have successfully completed installations, constituting 87.5 % of the total installed capacity in the community. Despite benefiting from national subsidies, these installations are not entirely cost-free. The payback period, which refers to the time required to recover the investment, is calculated based on various factors, including the 8 kW capacity, a subsidy period of 20 years, zero self-consumption rate, and full payment for the installation. The payback period serves as a theoretical calculation that considers factors such as the reduction in electricity costs or other forms of economic returns. The following example presents a theoretical calculation of the payback period, considering the unit price of household PV installation based on the market installation price in the L community in Nanjing. The power generation data mentioned in the analysis are obtained from the PV market, and specific details are suggested in Table 2.

**Table 2:** Historical change in the payback period of household rooftop PV investments.

Time	Installation price (Watt/yuan)	Total price (yuan)	On-grid price (kwh/yuan)	Subsidy price (kwh/yuan)	Cost recovery capacity (kwh)	Payback period (year)
2016	12	96000	0.391	0.42	118372	15.14
2017	10	80000	0.391	0.42	98644	12.49
2018.1–2018.5	8	64000	0.391	0.37	84100	10.57
2018.6–2018.12	8	64000	0.391	0.32	90014	11.35
2019	5	40000	0.391	0.18	70053	8.75
2020	4	32000	0.391	0.08	67941	8.47
2021	3.8	30400	0.391	0.03	72209	9.02
2022	3.8	30400	0.391	0	77749	9.74

**Fig. 3:**
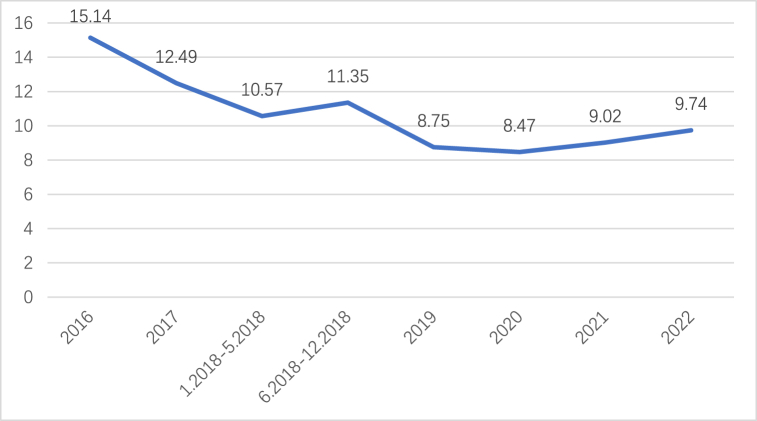
Rooftop PV payback period.

The payback period of a rooftop PV system varies in response to fluctuations in the system's cost and the subsidies available. It does not consistently decrease when the self-consumption rate is zero. Assuming a constant rooftop PV cost of 3.8 yuan per watt beyond 2022, the payback period stabilizes at 9.74 years, as revealed in Fig. 3.

#### 3.3.2 Household self-consumption rate

Rural electricity consumption in China has shown consistent growth, reaching 948.287 billion kilowatt-hours (kWh) by 2019. Provinces and cities with the highest rural electricity consumption in China include Jiangsu, Guangdong, and Shanghai. Jiangsu's electricity consumption gradually increased from 183.619 billion kWh in 2015 to 194.911 billion kWh in 2019 [35]. The average electricity consumption per household has also increased. Variations in daily electricity consumption among different households carry implications for the self-consumption ratios of rooftop PV systems, subsequently influencing their revenue potential.

A rooftop PV system's theoretical payback period can be calculated based on different self-consumption ratios. For example, an 8 kW system with a rooftop PV cost of 3.8 yuan per watt in 2022 and full payment installation would amount to 30,400 yuan. The minimum warranty period for rooftop PV products in the market is 20 years, and the theoretical power generation value per kilowatt of rooftop PV is 3.5 kWh. The total annual power generation would be 10,080 kWh, considering the self-consumption ratio as the independent variable. The electricity tariff is computed using the residential tariff for domestic electricity in Nanjing. The return on investment (ROI) for the rooftop PV system is calculated as follows: [Self-consumption PV power * (residential electricity price + PV subsidy) + residual on-grid power * (0.391 + PV subsidy)]/Installation cost. The payback period can be obtained as 12.96 times the reciprocal of the ROI, as outlined in Table 3.

**Table 3:** Payback period of rooftop PV with different self-consumption ratios.

self-consumption ratio	Self-consumption of electricity (kwh/year)	Reduction of electricity costs (yuan)	Annual On-grid return revenue (yuan)	Total revenue (yuan)	return on investment ratio (%)	Investment payback period (years)
0 %	0	0	3941	3941	12.96	9.74
10 %	1008	533	3547	4080	13.42	9.41
20 %	2016	1065	3153	4218	13.88	9.10
30 %	3024	1611	2759	4370	14.38	8.78
40 %	4032	2194	2365	4559	15.00	8.42
50 %	5040	2837	1971	4808	15.82	7.98
60 %	6048	3672	1577	5249	17.27	7.31
70 %	7056	4506	1182	5688	18.71	6.75
80 %	8064	5341	788	6129	20.16	6.26
90 %	9072	6176	394	6570	21.61	5.84
100 %	10080	7011	0	7011	23.06	5.47

The payback period of a rooftop PV system is directly impacted by the household's electricity self-consumption ratio. At a 0 % self-consumption ratio, the payback period is 9.74 years, whereas at a 100 % self-consumption ratio, the payback period reduces to 5.47 years. This signifies a reduction of 4.27 years, representing a 44 % decrease compared to the 0 % self-consumption ratio. When comparing the residual power on-grid model with the full self-consumption model, an 8 KW rooftop PV system generates an average of 32 kWh per day. Considering households' average daily electricity consumption at 8 kWh, surplus power is evident in the full self-consumption model. However, the full-power on-grid model yields the lowest benefits and is less conducive to recovering the investment cost.

## 4 Research results

### 4.1 Rural residents' type and number of rooftop PV

Based on the source of rooftop PV information and whether to consult others to make decisions, rural residents can be divided into two categories: independent decision-making and consulting decision-making, as shown in Table 4.

**Table 4:** Rural residents' type and number of rooftop PV.

Rural residents' Type			Quantity
Independent determination type	Type I.	Professional independent decision-making	1
	Type II.	Salesperson Promotion - Geographical Relationship (same/non-same village salesperson)	14
	Type III.	Salesperson promotion-cooperative business (PV users not in the same village)	6
Consultative- determination type	Type IV.	Salesperson Promotion-Geographical/blood relationship (PV users in the same village)-consult with independent decision-making users to make decisions	19

Type I residents predominantly rely on personal expertise and direct contact with rooftop PV companies to facilitate the installation of rooftop PV systems. Conversely, residents of other types acquire PV-related information through various marketing channels. Salespersons establish acquaintance and trust with residents through blood ties, geographical proximity, or business connections. These relationships, encompassing factors such as acquaintance, trust, co-career, same-village associations, and blood ties, foster a sense of "common community identity." In traditional trust systems, a preference exists for trust within "natural" groups, often referred to as an ethical "acquaintance society" [36]. As interpersonal interactions increase, there is a greater likelihood of mutual trust. However, with the acceleration of social mobility and a decline in interaction frequency, traditional boundaries defined by blood ties, kinship, and occupation gradually diminish in significance. Trust is more likely to emerge among individuals who perceive themselves as part of the same group (Mark Granovetter, Jiade Luo & Shuixiong Wang, 2019). The scope of "common community identity" expands accordingly, and if trust is not reciprocated or is betrayed, corresponding penalties come into effect. These penalties encompass potential economic losses as well as reputational damage.

Among the 40 households in the L community that have installed rooftop PV systems, only one household belongs to the acquaintance trust category. Within the framework of acquaintance trust, residents receive market information regarding PV company costs and knowledge of national PV subsidy policies through trustworthy salespersons.

### 4.2 Information sources provide different trust

The sources of rooftop PV information align with Granovetter's typology of Trust. The Trust types associated with different rooftop PV information sources can be categorized into three groups.

Drawing from rational choice theory, market trust is rooted in evaluations of economic benefits. It signifies the trust placed in market entities such as rooftop PV companies and their technological offerings.

Moreover, acquaintance trust is established through personal relationships, group and network affiliations, and adherence to shared norms. This form of trust encompasses the trust placed in rooftop PV company salespeople, existing rooftop PV users, and the Community Council.

Government trust pertains to the confidence placed in the rooftop PV subsidy policy implemented by the government, as demonstrated in Table 5.

**Table 5:** Trust types of different rooftop PV information sources.

Trust Type	Market Trust	Acquaintances Trust	Government Trust	Other villagers trust
Sources of trust				
Expertise	✓	/	/	/
Rooftop PV companies and salesman	✓	✓	/	/
Rooftop PV users	/	✓	/	✓
Community Council and Staff	⍻	⍻	⍻	✓
Rooftop PV subsidy policy	✓	/	✓	⍻

Notes: “✓” indicates that such trust type is available and provided; “⍻” indicates that such trust type is available but ultimately not provided; “/” indicates that such trust type is not available.

In Table 5, Community Council, staff, and rooftop PV subsidy policies play an indispensable role in shaping residents' trust thresholds. The Community Council and staff are pivotal in determining residents' trust threshold by offering market and industry assessments, being integral parts of residents' social networks, and serving as representatives of the government, acting as integrators of various trust types. Although the Community Council opts not to provide explicit recommendations on rooftop PV issues, it does not mean that market representatives replace them.

On the one hand, rural residents may harbor a certain level of distrust towards the ruling party, posing risks of controversy or misunderstandings when the Community Council provides advice [37]. On the other hand, Community Council staff aim to avoid making mistakes or misrepresenting party positions when offering specific recommendations [38]. This does not diminish the significance or relevance of the Community Council in rooftop PV matters. By embodying the identity of the nation and the government, the Community Council provides market trust and government trust to residents. However, due to the influence of their political identity, exercising all types of trust concerning rooftop PV issues proves more challenging for Community Council staff.

### 4.3 Survey results and analysis

The extent of rural residents' participation in the fair transition to green energy is presented in Table 6.

**Table 6:** The extent of rural residents' participation in the fair transition to green energy.

Participation	Highly Engaged (%)	Moderate participation (%)	Limited Participation (%)
population sample	35	45	20
under the age of 30	40	40	20
Age 31-50	35	50	15
over the age of 51	30	45	25

In Tables 6 and in the overall sample, 35 %, 45 %, and 20 % of the rural residents show a high, moderate, and limited degree of participation, respectively. According to the analysis of different age groups, among residents under the age of 30, 40 %, 40 %, and 20 % indicate high, moderate, and limited participation, while among residents aged 31–50 and over 51, participation levels vary. The main motives and interests of rural residents participating in green energy are listed in Table 7.

**Table 7:** Main motivations and interests of rural residents to participate in green energy.

main motives and interests	Environmental protection (%)	Energy conservation (%)	Economy Profit (%)
population sample	50	30	20
Gender: Male	45	35	20
gender: female	55	25	20
Education level: low	40	35	25
Education Level: Medium	50	30	20
Education Level: High	55	25	20

The main motivations and benefits of rural residents' participation in green energy, as displayed in Table 6, include environmental protection (50 %), energy conservation and emission reduction (30 %), and economic benefits (20 %). Gender and education level analyses indicate that women exhibit a stronger motivations in environmental protection, while men prioritize energy conservation and emission reduction as well as economic benefits. The level of trust of rural residents in different sources of information is illustrated in Table 8.

**Table 8:** Level of trust rural residents have in different sources of information.

Information Sources	High trust (%)	Moderate trust (%)	Low Trust (%)
Government Roof PV Subsidy Policy	40	45	15
Rooftop PV companies and salespeople	30	50	20
Roof PV users	25	55	20
Community Board and Staff	35	40	25
family and relatives	20	50	30
friends and neighbors	15	55	30

In Table 8, rural residents have a high degree of trust (40 %) in the government's rooftop PV subsidy policy, and the proportions of high and moderate trusts are 40 % and 45 %, respectively. Moderate trust is highest at 50 % for rooftop PV companies and salespeople, compared to 55 % for rooftop PV users. Community boards and staff are highly trusted by 35 % but low by 25 % of rural residents. In addition, family members and relatives, friends, and neighbors are also trusted to a certain extent as sources of information, but the degree of trust is relatively low.

The statistics of obstacles affecting rural residents’ participation in the transition to green energy are denoted in Table 9.

**Table 9:** Barriers to rural residents' participation in a transition to green energy.

Obstacles	Height Obstacles (%)	Moderate Handicap (%)	Low Handicap (%)
economic cost	45	40	15
lack of expertise	30	50	20
Unstable Policy Environment	35	45	20
Social acceptance is not high	20	50	30

Table 9 presents the obstacles rural residents face in participating in a fair energy transition. The most significant obstacle identified is the economic cost, with 45 % and 40 % of rural residents indicating a high and moderate obstacle.

## 5 Conclusion and discussion

### 5.1 Discussion

The acceptance of green energy products requires overcoming the economic and trust thresholds. Within the economic threshold, considerations include the economic benefits and time investment associated with rooftop PV. Concurrently, the trust threshold entails trust in the sources of rooftop PV information. The trust threshold acts as a facilitator in fully traversing the economic threshold. This study draws the following conclusions by analyzing the mechanisms involved in rural residents' engagement in a transition to green energy.

The economic threshold and trust threshold are intricately interconnected. Rural residents, leveraging acquaintances based on familial, geographic, and professional ties, gain access to information on the revenue potential of the rooftop PV market and government PV subsidy details. This enables them to enter the economic threshold of assessment. Rural residents perceive rooftop PV as financially viable by considering household electricity consumption and economic benefits. However, to fully cross the economic threshold, support from acquaintances, market entities, and government policies in the form of acquaintance, market, and government trust, respectively, is essential.

The acquaintance economy plays a pivotal role in the initial phase of establishing the rural rooftop PV market. Identity brings trust [39]. However, it has inherent limitations when promoting a transition to green energy and achieving carbon reduction goals. Within the L community, information dissemination concerning rooftop PV primarily occurs among residents within each natural village based on geographical or familial connections. Subsequently, those who install rooftop PV after consulting these independent residents become consultative determination users, representing a form of acquaintance economy diffusion. Rural society is characterized by limited mobility, while modern society is highly mobile, with individuals inhabiting mobile spaces [40].

The withdrawal of rooftop PV subsidy policies has led to a decline in residents' economic benefits, illustrating that the withdrawal of government PV subsidies has indirectly affected market trust and the government's trust in rural residents. Within the economic threshold, theoretical calculations have confirmed that as rooftop PV installation costs decrease, government subsidies gradually diminish, aligning with changes in rooftop PV costs. However, the government must pay close attention to the market trust and government trust associated with the policies it implements. From an economic standpoint, government subsidies should not be eliminated. The government can regularly release information demonstrating the economic benefits to mitigate the uncertainty surrounding economic returns for rooftop PV among rural residents. Simultaneously, targeted public information and services ought to be tailored to support the operation and maintenance of rooftop PV, thereby bolstering rural residents' confidence in installation and facilitating their crossing of the trust threshold.

This study is conducted in the L community, where rural residents self-fund their rooftop PV installations. It underscores the importance of considering the dual thresholds of the economy and trusts to effectively and efficiently promote a transition to green energy, achieve sustainability in suburban counties, and ultimately fulfill carbon reduction goals.

This study denotes that economic and trust factors significantly influence the participation of rural residents in the green energy transition, particularly in the case of rooftop PV projects in the suburban areas of Nanjing. This discovery aligns with research results from other countries and regions. For instance, in Germany, Seel et al. (2014) indicated that government subsidies and electricity price guarantee mechanisms remarkably increased residents' motivation to install rooftop PV systems, thus accelerating the green energy transition [41]. In South Korea, Kim et al. (2021) found that the prevalence of rooftop PV systems in social networks and neighboring communities positively influenced individual decisions to install, aligning with Granovetter's embeddedness theory and this study's results [42]. Additionally, in California, USA, Zheng et al. (2020) revealed the crucial role of government incentive measures in reducing initial investment costs and shortening the payback period by analyzing the economic feasibility of rooftop PV projects [43]. This also supported this study's finding that government policy support played a crucial role in promoting the participation of rural residents in the green energy transition. In a study in India, residents in rural areas generally perceived the high economic cost of green energy technologies as a significant factor affecting their participation in the transition [44]. Therefore, reducing the economic burden on rural residents to participate in the green energy transition was crucial to encouraging more widespread engagement. Moreover, low social acceptance was a significant barrier to rural residents' participation in the green energy transition. Related studies suggest that in some European countries, rural residents hold reservations about emerging green energy technologies, primarily due to a lack of understanding of these technologies and the convenience of traditional energy sources [45]. Thus, increasing rural residents' awareness of green energy, strengthening the promotion of its advantages, and enhancing their acceptance of the green energy transition can be beneficial. However, it is essential to note that, although the above studies emphasize the importance of economic incentives and social networks in driving the green energy transition, cultural differences and region-specific factors present different impact patterns. For example, compared to rural communities in the suburban areas of Nanjing, urban residents in Europe and North America may place more emphasis on environmental protection and sustainable development values rather than just economic benefits. Therefore, when designing and implementing relevant policies, these differences need to be taken into account. In summary, this study provides insights into the motivations and obstacles for rural residents in Nanjing to participate in the green energy transition. Moreover, through comparison with other countries and regions, the similarities and differences in different social and cultural backgrounds are highlighted. This offers crucial policy insights for advancing the global green energy transition.

### 5.2 Research conclusion

This study shows that the knowledge level of rural residents is related to their participation, and younger and better-educated residents are more actively involved in the green energy transition, supporting the study hypothesis. Simultaneously, they also have high levels of trust in government policies and social networks, further supporting the hypothesis. According to the above research results, the following conclusions can be drawn. The knowledge level of rural residents, government policy support, and social networks all influence their level of participation in a fair energy transition. Rural residents with higher knowledge levels are more inclined to participate actively, government policy support can encourage their participation, and extensive social networks help rural residents access information and resources, thus enabling them to participate more actively in the green energy transition.

Conclusively, the examination of rural residents' participation in the green energy transition in Nanjing, China underscores the significance of analyzing communities. Participation is influenced by a blend of social, economic, and policy elements, with key factors being social networks, trust, economic incentives, and governmental policies. It is imperative to comprehend and tackle these aspects at the community level for the effective adoption of sustainable energy practices in rural regions.

## Data availability statement

The data that has been used is confidential.

## CRediT authorship contribution statement

**Ling Li:** Methodology, Investigation, Data curation. **Jintu Gu:** Conceptualization, Writing – review & editing. **Di Wu:** Formal analysis, Visualization, Writing – review & editing. **Abdul Rasool khoso:** Visualization, Writing – review & editing.

## Declaration of competing interest

The authors declare that they have no known competing financial interests or personal relationships that could have appeared to influence the work reported in this paper.
