# Supplementary material for: Mechanism analysis of rural residents' participation in green energy transition: A community-level case study in Nanjing, China
Source: Heliyon. 2024 Jul 2;10(13):e33951. doi: 10.1016/j.heliyon.2024.e33951 (PMC11283147; doi:10.1016/j.heliyon.2024.e33951)
Supplement: Multimedia component 1 [file mmc1.docx]

Dear Participant:

This study aims to investigate ‘**Mechanism Analysis of Rural Residents' Participation in Green Energy Transition’**. Your participation is voluntary, and you may withdraw from the study at any time without penalty.

The research process includes the following:

1. The researcher will collect personal data from the participants, including but not limited to age, gender, and educational level.

2. The researcher will collect data related to the participants' rooftop photovoltaic (PV) systems, including but not limited to monthly earnings, monthly electricity production from PV, and installation costs.

3. The researcher will conduct case interviews to collect information about the participants' sources of information regarding rooftop photovoltaic (PV) and the factors considered in their installation decisions.

Your personal information will be kept confidential and used only for the purposes of this study. Upon completion of the research, all data linking to your personal identity will be destroyed.

**Interview Questions**

1.Personal Background Information:

·What is your age?

·What is your gender?

·What is your educational level?

·How many people reside in your household?

2.Rooftop Photovoltaic (PV) System Information:

·When was your rooftop photovoltaic (PV) system installed?

·What was the total cost of installing your rooftop photovoltaic (PV) system?

·Approximately how much electricity does your rooftop photovoltaic (PV) system generate per month?

·How much revenue do you earn from your rooftop photovoltaic (PV) system per month?

·Do you incur any maintenance costs for your rooftop photovoltaic (PV) system? If yes, what is the approximate amount?

3.Information Sources and Installation Decision Factors:

·Through which channels did you acquire information about rooftop photovoltaic (PV) systems?

·What were the main factors you considered when deciding to install a rooftop photovoltaic (PV) system?

·Were there any particular factors that you prioritized during the decision-making process?

·Did you seek advice from professionals or friends and family? If yes, how did their input influence your decision?

·What do you believe were the decisive factors that led you to install a rooftop photovoltaic (PV) system?

Thank you for your participation.Best wishes for a happy and comfortable life.
